# Research Progress and Application Scenarios of Wire + Arc Additive Manufacturing: From Process Control to Performance Evaluation

**DOI:** 10.3390/mi16070749

**Published:** 2025-06-25

**Authors:** Chun Guo, Qingcheng Lin, Ruizhang Hu, Suisong Wu

**Affiliations:** 1 College of Intelligent Manufacturing, Anhui Science and Technology University, Chuzhou 233000, China; 17333218178@163.com (Q.L.); hurzh@ahstu.edu.cn (R.H.); 2 College of Mechanical Engineering, Anhui Polytechnic University, Wuhu 241000, China; wuss@enigmaautomation.com

**Keywords:** WAAM, microstructure, mechanical properties, improve methods

## Abstract

In recent years, with the innovation and continuous development of additive manufacturing technology, research on wire arc additive manufacturing technology (WAAM) has become increasingly common and in-depth in the chemical industry, mold manufacturing, and other fields. Therefore, it has attracted the attention of many universities, research institutes, and aerospace industries, conducted in-depth research on WAAM technology, and achieved certain research results. This paper briefly summarizes the current research status of arc additive manufacturing technology and summarizes the application status of WAAM technology in product development, personalized customization, traditional process replacement, “material–structure–function” integration, mold repair, etc. WAAM technology has huge development potential and good application prospects. In the future, arc additive manufacturing will develop in the direction of intelligence and high precision.

## 1. Introduction

Additive manufacturing (AM), also known as 3D printing technology (3D printing), is a technology that combines CAD/CAM design programming software and modern processing and manufacturing technology to accumulate materials layer by layer into required physical objects [1,2,3]. Compared to traditional subtractive manufacturing (e.g., cutting), additive manufacturing (AM) adopts a “bottom-up” approach that shortens the production cycle, reduces processing time, enhances flexibility, and increases material utilization—particularly since some materials can be reused. As a result, AM significantly lowers manufacturing costs and conserves both labor and material resources [4,5]. Therefore, AM has great advantages, especially in the manufacture of large-scale components with complex shapes [6,7,8].

**Figure 1:**
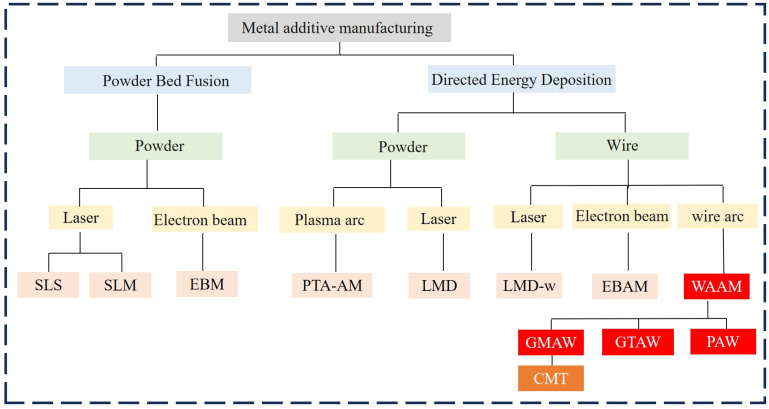
Additive manufacturing technology classification.

In recent years, AM technologies have rapidly evolved and have been categorized into various types based on material and process principles. Figure 1 shows a classification map of metal additive manufacturing. Metal additive manufacturing (MAM) is a crucial branch, including powder bed fusion (PBF), directed energy deposition (DED), and wire arc additive manufacturing (WAAM) [9,10]. Among additive manufacturing techniques, powder bed fusion (PBF) processes—such as selective laser melting (SLM) and electron beam melting (EBM)—offer high precision and excellent surface quality [11,12]. However, they are constrained by limited build sizes and high equipment costs. In contrast, directed energy deposition (DED) enables higher deposition rates but requires complex laser or electron beam control systems [13]. WAAM distinguishes itself with its significantly higher deposition rates, lower cost, greater scalability for large parts, and simpler system setups, making it highly attractive for industrial applications. The comparison of several metal additive manufacturing process methods is shown in Table 1.

(1) WAAM has a high degree of freedom. The driving systems used in laser and electron beam additive manufacturing are primarily multi-axis machine tools, typically employing Cartesian three-axis translation to achieve bottom-up material deposition. In contrast, arc-based additive manufacturing commonly utilizes multi-axis robotic arms equipped with external axes. Positioning machines and gantry frames can realize the manufacturing of a high degree of freedom, high flexibility, and any complex spatial path.
(2) WAAM is suitable for the forming of large and complex structural parts, while lasers and electron beams are mostly operated in closed rooms, and the forming environment is limited, which makes it difficult to meet the manufacturing requirements of large metal components [20,21,22].
(3) WAAM is also suitable for the repair of certain simple components.

**Figure 2:**
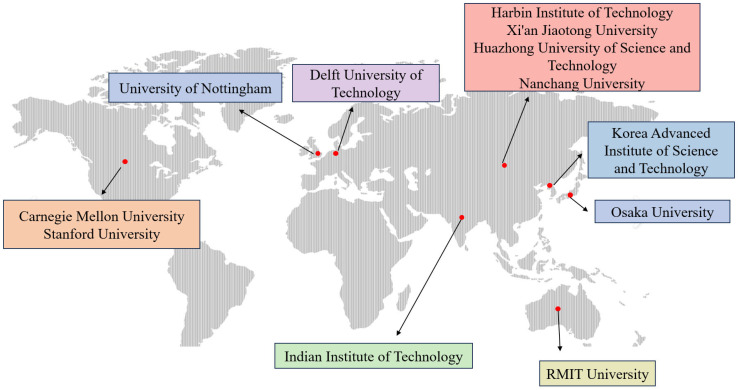
Main research institutions of WAAM around the world.

Wire arc additive manufacturing (WAAM) has attracted significant attention in aerospace, machinery manufacturing, shipbuilding, and other industrial sectors. China’s “Made in China 2025” strategic initiative also clearly identifies additive manufacturing equipment and materials as one of the key development directions of the manufacturing industry, giving WAAM very broad development prospects [23]. At present, as shown in Figure 2, many R&D institutions have achieved relatively mature applications. Compared to laser and electron beam additive manufacturing, which primarily employs multi-axis machine tools to achieve layer-by-layer deposition through Cartesian motion, WAAM typically utilizes multi-axis robotic arms equipped with external axis positioners and gantry frames [24,25,26]. This configuration enables the fabrication of components with high degrees of geometric freedom, enhanced flexibility, and the ability to follow complex spatial trajectories. Historically, as early as the early 20th century, Baker et al. in the United States first manufactured metal objects by arc surfacing welding and simultaneously applied for a patent. In 1983, German researchers proposed using metal wire additives and submerged arc welding to fabricate large and complex metal parts. By the end of the 20th century, alongside rapid manufacturing research, WAAM technology began to attract significant interest [27,28]. In 2007, Cranfield University initiated systematic research on WAAM and applied the technology to the rapid fabrication of aircraft fuselage structures. Nowadays, with the rapid development of digitalization, informatization, and intelligent manufacturing, WAAM research has been greatly advanced in terms of forming control, performance optimization, and production efficiency [29]. Parallel to these advances, a new research paradigm is emerging that couples WAAM with data-centric and model-driven manufacturing concepts. In particular, physics-informed machine-learning (ML) algorithms are now being trained on high-frequency process data (current, voltage, acoustic emission, melt-pool imaging) to predict bead geometry and microstructure in real time [30,31]. Digital twin frameworks that integrate machine learning (ML) models with finite element thermal–mechanical simulations enable “what-if” analyses prior to deposition, thereby reducing trial-and-error iterations on the shop floor [32,33]. These developments signal a shift from purely empirical optimization toward AI-driven, standards-compliant WAAM ecosystems capable of closed-loop quality assurance [34].

Several review articles on WAAM have appeared during the last decade. Early studies [35,36,37,38] established the foundational process science, while more recent reviews have explored specific aspects such as path-planning algorithms, in-situ monitoring and control, and alloy-specific developments in aluminum and titanium systems. Nevertheless, a comprehensive perspective that simultaneously integrates process optimization, real-time quality assurance, microstructure–property relationships, hybrid subtractive finishing, and emerging AI-driven standards remains lacking. The present review therefore seeks to close this gap by synthesizing the dispersed literature—particularly the surge of work published in 2023–2025—and by mapping unresolved scientific and industrial challenges to concrete research directions. Therefore, this paper systematically presents the current research status of WAAM technology, including process optimization, path planning, monitoring and control during the forming process, and post-processing techniques [39]. It further analyzes common defects such as porosity, residual stress and strain, and cracking, along with corresponding mitigation strategies. Additionally, it summarizes the current applications of WAAM in industry. Finally, this review points out the challenges and prospective future directions of WAAM, aiming to promote further development of this promising technology [40].

## 2. WAAM Technology

**Figure 3:**
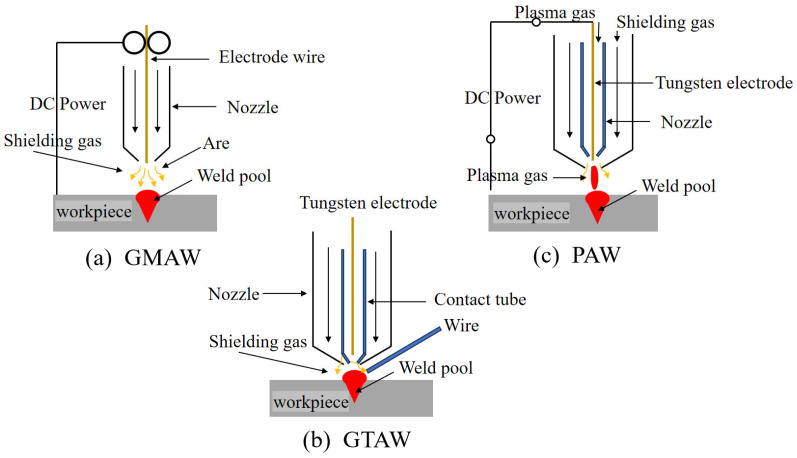
WAAM classification.

**Figure 4:**
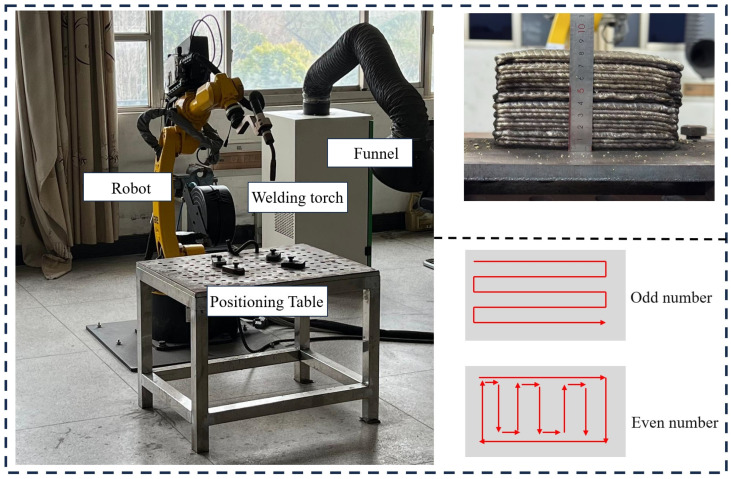
Wire arc additive equipment, path planning, and forming sample.

Figure 3 shows the schematic diagram of several common types of arc additive manufacturing. Generally, based on the nature of the heat source, additive manufacturing processes can be categorized into three types: gas metal arc welding (GMAW), gas tungsten arc welding (GTAW), and plasma arc welding (PAW). The energy source is typically combined with a six-axis manipulator or CNC machine tool that serves as the motion platform [41]. There are two types of typical gas protection devices commonly used: one is a closed gas chamber, which can provide good inert gas protection similar to the laser selective melting level, but the size of the closed gas chamber will limit the size of the manufactured parts [42]; the other is a partial gas protection device, usually installed at the welding torch, which only provides a good local inert gas protection atmosphere for the hot molten pool and the area near the molten pool during the accumulation process. This device is suitable for the manufacture of large-scale components [43]. The WAAM system mainly involves process planning, stacking, post-processing, etc. The parts formed by WAAM technology are composed of full weld metal, with uniform chemical composition and high density. The free-forming environment imposes virtually no limitations on part size, and its forming efficiency can reach several kilograms per hour, surpassing that of traditional casting and forging technologies. Moreover, it offers advantages over other additive manufacturing techniques in terms of advancement and productivity [44,45]. In contrast to traditional forging and casting methods, this process eliminates the need for molds, offers a shorter overall production cycle, and provides enhanced flexibility. It also supports digitalization, intelligent control, and concurrent manufacturing. Moreover, the resulting components often exhibit superior strength and improved toughness compared to monolithic forgings. Additionally, due to the repeated heating, quenching, and normalizing that occur during the layer-by-layer deposition, issues such as poor hardenability, macro segregation, and directional differences in mechanical properties—commonly seen in large cast components—can be effectively reduced [46,47]. WAAM technology also has many deficiencies. Because the arc is a flexible conductor, it is easily disturbed by the external environment, causing the arc to be unstable, resulting in reduced component forming accuracy and accumulation defects such as pores in the component, which lead to reduced component-forming quality [48,49,50,51]. At this stage, some scholars at home and abroad are also committed to researching the method of “additive-subtractive” composite manufacturing to improve the forming accuracy of components. Some scholars also use the method of plastic processing of components after arc additive manufacturing to eliminate the forming defects of components, and based on this, have developed an integrated equipment system for WAAM. The integrated arc addition and subtraction equipment of the integrated milling module, the arc gun, and the end mill for material reduction are, respectively, installed on two robots. The upper surface and side of the stacked metal are milled and cut to improve the forming accuracy of the stacked metal. Figure 4 shows a common laboratory arc additive device, including the scanning strategy and the shaped part diagram [52].

As aerospace, national defense, military, and other important technical fields have increasingly higher requirements for the cost, cycle, performance, and precision of expensive metal parts, WAAM has shown great promise in military, aerospace, and automotive markets due to its high flexibility, adaptability, and superiority. Because arc additive manufacturing technology can directly form metal parts, it has become a research hotspot in universities, research institutes, and large enterprises at home and abroad [53].

## 3. Research Status of WAAM Technology

**Figure 5:**
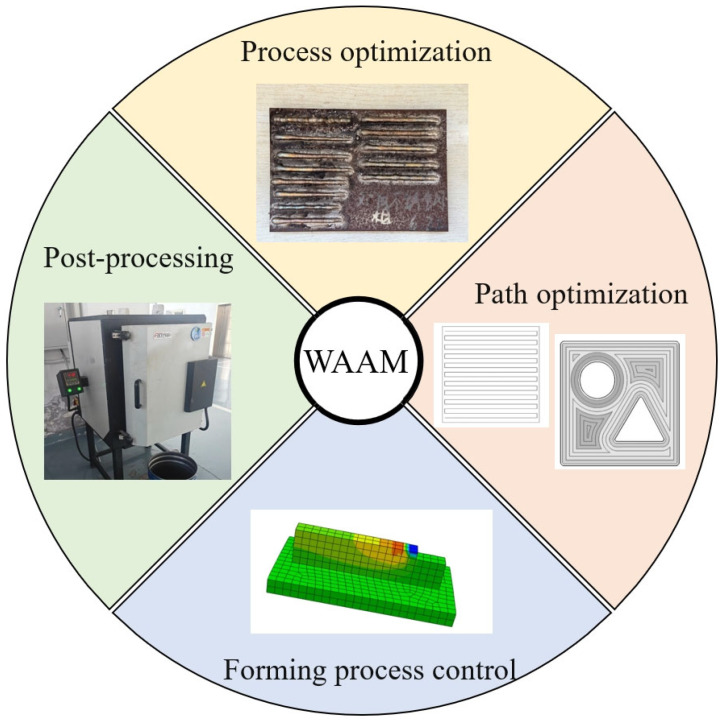
Research direction in recent years.

The origins of wire arc additive manufacturing (WAAM) can be traced back to 1925, when Baker and his colleagues at Westinghouse Electric first employed an electric arc as a heat source to fabricate metal parts through a layer-by-layer deposition process. However, due to the limitations of digital technologies at that time, this approach did not attract widespread research interest. It was not until the 1990s, with the significant advancements in computer systems and digital control, that WAAM began to receive growing attention and rapid development [54]. Over the past three decades, researchers worldwide have increasingly investigated WAAM, emphasizing critical factors such as dimensional accuracy and performance consistency—key evaluation metrics for arc-based additive manufacturing. During the WAAM process, materials undergo intense physical and chemical transformations, involving intricate metallurgical mechanisms and deformation behaviors, influenced by numerous variables [55,56,57]. Figure 5 shows the mainstream research direction. In recent years, universities and research institutes worldwide have concentrated their efforts on optimizing process parameters, path planning, real-time monitoring and control strategies, as well as post-processing techniques. These investigations aim to clarify the links between materials, processes, microstructures, and final properties, enabling more precise control over the forming quality.

### 3.1. Process Selection and Optimization of WAAM

Unlike laser- or electron beam-based additive manufacturing methods, arc additive manufacturing (WAAM) generates a relatively large molten pool. This pool becomes inherently unstable due to external disturbances such as cold feedstock and arc forces during deposition. To produce components with accurate geometry and desirable mechanical properties—collectively referred to here as “form”—the molten pool must exhibit stable and repeatable behavior throughout the process [58]. Therefore, selecting the appropriate process type and fine-tuning its parameters are fundamental to achieving high-quality WAAM parts. WAAM can generally be classified into two categories based on the wire feeding approach. The first utilizes coaxial wire feeding with consumable electrode arcs. This includes conventional arc welding methods such as Metal Inert Gas (MIG) welding and advanced processes like Cold Metal Transfer (CMT). The second approach utilizes off-axis wire feeding based on Plasma Arc Welding (PA), which can also be substituted with Tungsten Inert Gas (TIG) welding [59,60,61].

From the perspective of process optimization, early research primarily employed empirical methods—testing various combinations of welding techniques and filler wire types to identify key process parameters. Key process parameters include welding speed, wire diameter, wire feed rate, stick-out length, interlayer temperature, current, voltage, as well as the type and flow rate of shielding gas. The goal has been to define how these parameters correlate with part quality. More recent studies have adopted a predictive approach, employing regression models to correlate process parameters with bead geometry, thereby identifying optimal parameter sets to ensure dimensional accuracy and performance consistency of WAAM-fabricated components. Research has also revealed that TIG-based WAAM, due to the non-coaxial alignment of arc and filler wire, faces challenges when dealing with complex deposition paths [62]. The relative phase between wire feeding and the deposition direction is governed by the motion system, introducing additional complexity to process control. Conversely, MIG-based WAAM features a coaxial arrangement of arc and wire, which simplifies operation by eliminating the phase dependency. This results in faster deposition rates and improved accessibility during fabrication [63,64,65].

To further enhance WAAM, Fronius developed Cold Metal Transfer (CMT) technology—a variant of MIG/MAG welding characterized by ultra-low heat input, spatter-free droplet transfer, and arc stability. These traits make CMT particularly suitable for additive manufacturing. For example, Kazanas from Cranfield University applied CMT to carbon steel and aluminum alloys, leveraging its low heat input for process refinement [66,67]. Their study also diverged from traditional vertical torch deposition by introducing all-position welding, enabling the additive fabrication of sloped and enclosed thin-walled structures. This approach eliminated the reliance on positioners for building complex geometries and significantly improved the consistency and repeatability of WAAM processes.

### 3.2. WAAM Technology Path Planning

WAAM technology involves complex and variable process parameters, which demand precise path planning, especially for workpieces with intricate geometries. Therefore, in order to make arc additive manufacturing reach the level of economical, efficient, and intelligent, it is necessary to jointly optimize the process parameters and path planning to meet the shape-related requirements of the formed part. Over the past few years, an increasing number of research institutions worldwide have focused on path planning strategies for arc-based additive manufacturing [68]. This body of research primarily focuses on two core areas: first, optimizing toolpaths based on the model’s structural features to improve melt track geometry and surface precision; and second, optimizing thermal distribution during the deposition process to minimize residual stresses and reduce overall part distortion. A notable contribution comes from the University of Wollongong in Australia, where researchers approached WAAM path planning by proposing an innovative CAD model slicing technique to better support these objectives. The method decomposes a 2D geometry into a series of convex polygons, then applies an optimized scan direction to each convex polygon, combining zigzag and contour-mode scanning strategies to generate a continuous path. Finally, all independent subpaths are connected to form a closed curve [69]. This strategy satisfies the design requirements for the straightforward implementation of WAAM, minimizing the number of arcing/extinguishing points and achieving high surface precision [70,71]. In addition, in order to improve the quality of surface forming, the expert team proposed a more adaptable bone offset path planning method based on the transformation of the central axis to eliminate internal vacancies and to automatically generate a path with 100% coverage for any complex path. Building on the bone offset path, this method further proposes a bone non-parallel offset path that enables adaptive path planning based on the cross-sectional shape, achieves compact and gap-free deposition, improves the geometric accuracy of the workpiece, and reduces material consumption by up to 27% or more. This series of studies has made a great contribution to the optimization of path planning for arc additive manufacturing. At Nanjing Inigma Process Automation Technology Co., Ltd. (Nanjing, China), the engineering team has created lungoPNT—an intelligent path optimization tool that utilizes a layered slicing algorithm tailored for STL model processing. This software enhances slicing paths by analyzing the structural attributes of the workpiece model. It intelligently detects specific features in the digital model—such as overlaps, corners, thin sections, and narrow gaps—that may necessitate tailored processing. Utilizing its built-in algorithms, lungoPNT automatically refines the printing sequence, arc initiation and termination, path offsets, and infill strategies. These optimizations significantly reduce defect rates in printed components and boost manufacturing efficiency.

Despite these encouraging demonstrations, several unresolved challenges remain. (1) Most reported algorithms—including the convex-polygon and bone-offset strategies cited above—assume a quasi-planar build plane; they struggle with steep overhangs, locally negative Gaussian curvature, or multi-directional deposition demanded by conformal-cooling channels and lattice infills [72]. (2) Current work typically targets either surface fidelity or thermal uniformity, whereas industrial adoption requires multi-objective strategies that also minimise arc on/off events, wire-feed reversals, and total build time [73]. Multi-criteria genetic-algorithm or reinforcement-learning approaches show promise, but benchmark data sets for fair comparison are lacking.

### 3.3. Monitoring and Control Optimization of Forming Process in WAAM Technology

Due to its reliance on arc welding techniques, WAAM inherently faces challenges such as a broad heat-affected zone, relatively low heat flux density, and intense heat input. During continuous deposition, the combination of high-energy arc sources and low heat dissipation efficiency leads to excessive heat accumulation. This thermal buildup significantly hinders the control of geometric shape and dimensional accuracy in the fabricated workpiece [74,75]. Therefore, only by realizing fully intelligent and digital monitoring and control in the arc additive manufacturing process can the entire arc additive manufacturing be more stable, thereby increasing the success rate of material forming and improving integrity of forming. Current research on the WAAM process primarily focuses on two key aspects of monitoring, feedback, and control: first, real-time tracking and regulation of additive process parameters; and second, shape monitoring and control utilizing vision system technology [76]. Over the past few years, there has been limited domestic research on the real-time monitoring and regulation of arc additive manufacturing process parameters. At Cranfield University, both a low-frequency system for real-time data acquisition and a high-frequency arc monitoring system have been developed. The low-frequency system tracks key parameters such as robot end position, current, voltage, wire feed speed, and layer height, with an adjustable monitoring frequency of up to 20 Hz, primarily used for compensation of compartment height during the printing process. The high-frequency system, on the other hand, monitors parameters like current, voltage, wire feed speed, and temperature, with a maximum frequency of 2000 Hz, enabling detailed tracking of droplet transitions and the stability of process parameters.

Current sensing architectures can be categorized broadly into scalar channels (current, voltage, wire-feed speed) and imaging channels (coaxial melt-pool vision, laser profilometry, infrared thermography). However, their integration remains ad hoc and specific to individual laboratory setups [77,78]. Moreover, translating raw data into actionable quality metrics remains an open challenge. Present practice relies on heuristic thresholds—for example, voltage windows or pixel-intensity bands—rather than physics-informed or probabilistic predictors of bead morphology and porosity. Robust machine-learning models would require large, labelled data sets, but these are scarce and often proprietary. Creating publicly accessible “defect atlases” for WAAM, akin to weld-quality image repositories in conventional arc welding, would facilitate algorithm training and benchmarking.

### 3.4. Wire Arc Additive Technology Performance Testing and Analysis

(1) Micro-organizational analysis

After wire cutting, inlaying, and polishing, the prepared samples are generally observed by optical microscope and scanning electron microscope to observe their microstructure. The microstructure of WAAM-fabricated components is fundamentally influenced by process parameters, including heat input, deposition strategy, interpass temperature, and cooling rate. Due to the high deposition rates and multiple thermal cycles intrinsic to WAAM, the as-built microstructure typically exhibits significant anisotropy and hierarchical grain structures [79].

**Figure 6:**
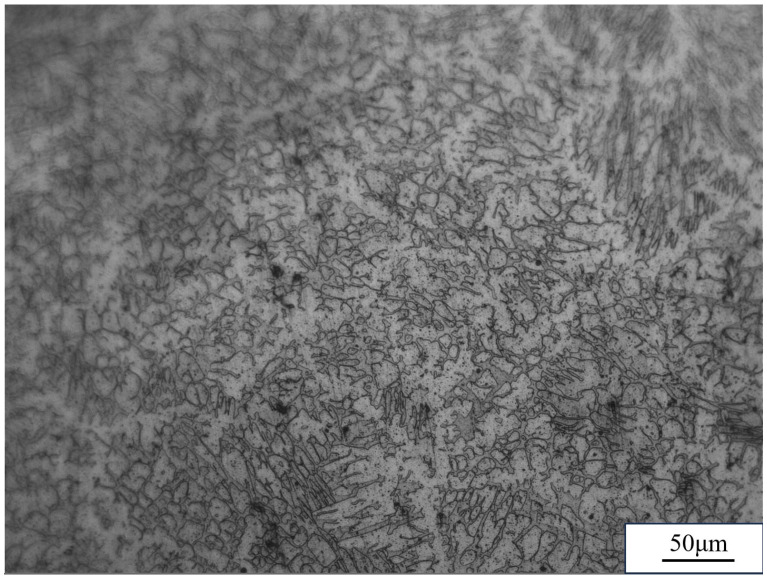
Microstructure of arc additive manufacturing technology based on CMT.

In GTAW-based WAAM, fine equiaxed grains are often observed near the fusion boundary, transitioning to coarser columnar grains oriented along the build direction as a result of directional solidification. In contrast, GMAW and CMT-based processes, owing to their inherently higher deposition rates and relatively lower arc stability, often promote the formation of larger, more epitaxially grown columnar grains. PAW-based WAAM, benefiting from its concentrated energy input, tends to yield finer dendritic structures compared to traditional GMAW. When compared with powder bed fusion (PBF) processes, WAAM generally produces larger grain sizes and lower cooling rates, leading to inferior inherent microstructural refinement. However, it excels in achieving dense structures with fewer internal defects such as porosity, provided optimal process parameters are maintained. Figure 6 shows the longitudinal microstructure spectra of ER2594 duplex stainless steel prepared by arc additive manufacturing based on CMT. It can be seen that the samples are mainly composed of black and white austenite (γ) and ferrite (δ).

(2) Mechanical property testing and analysis

Mechanical performance evaluation is critical for WAAM components intended for load-bearing applications; it includes microhardness, wear resistance, tensile impact performance, and so on. The tensile properties of WAAM-fabricated parts are highly dependent on build orientation, owing to anisotropic grain structures and the presence of potential inter-layer defects [80].

**Figure 7:**
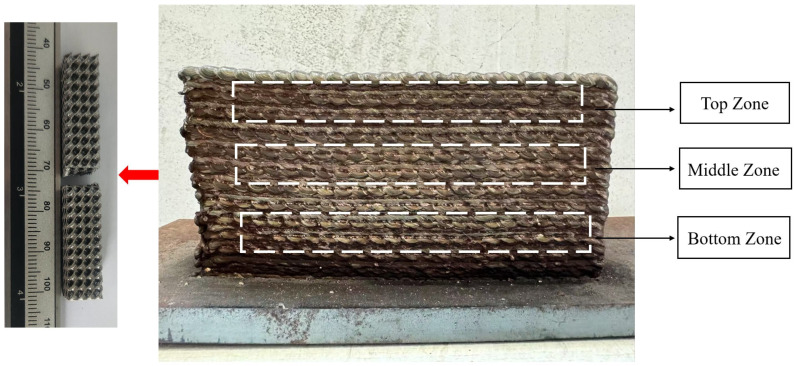
Schematic diagram of sample cutting.

In general, WAAM-fabricated components exhibit lower yield strength and tensile strength in the build direction compared to the transverse direction, attributed to columnar grain morphology and weaker interpass bonding. Post-processing techniques such as interlayer rolling, heat treatment, and hot isostatic pressing (HIP) have been shown to mitigate these discrepancies and enhance isotropy. Comparative studies have demonstrated that while the as-built mechanical properties of WAAM parts are generally inferior to those produced by PBF in terms of ultimate tensile strength and fatigue resistance, they surpass those of DED-built components in terms of toughness and ductility, especially when optimized processing conditions are employed. For the same material, different types of arc additive manufacturing yield varying structures. For example, wire arc additive manufacturing using CMT and GMAW technologies has been applied to SS316L material [81,82]. Figure 7 is a schematic diagram of sample cutting, the specific results are shown in Table 2 and Table 3, but they all follow the same pattern: the average microhardness and impact performance increase near the substrate. Samples were taken from the bottom, middle, and top of the prepared alloy for microhardness and tensile impact tests. The results show that compared to SS316L alloys produced by cold metal transfer arc additive manufacturing (CMT), those made using gas metal arc welding (GMAW) have slightly lower hardness but significantly increased tensile strength and yield strength.

### 3.5. WAAM Technology Post-Processing

The microstructure of components fabricated by WAAM is highly sensitive to the complex and cumulative thermal history inherent in the process. As the moving arc heat source induces repeated thermal cycling, localized overheating and pronounced thermal gradients result in coarse columnar grains, heterogeneous phase distribution, and residual stresses. These microstructural features, in turn, affect key mechanical properties such as strength, ductility, toughness, and fatigue resistance. Therefore, post-processing is not simply an auxiliary step, but rather a critical enabler for tailoring final performance and ensuring functional reliability in WAAM-produced components. Among the most commonly employed post-processing methods are heat treatment, rolling, and subtractive machining, each targeting specific process-induced issues. Heat treatment, including stress relief annealing and solution-aging protocols, is widely applied to reduce residual stress and to promote recrystallization or phase homogenization. However, the effectiveness of thermal treatment alone is often limited, especially for alloys prone to grain coarsening at elevated temperatures. Thus, researchers have turned to mechanically assisted post-processing techniques to further refine the microstructure and improve dimensional accuracy.

At Huazhong University of Science and Technology, for example, an integrated arc micro-casting and rolling process has been proposed, wherein micro-scale rolling is applied to the high-temperature zone immediately after deposition. This approach not only disrupts epitaxial grain growth but also promotes plastic deformation, resulting in grain refinement and improved tensile properties. Similarly, researchers at the Korea Institute of Science and Technology developed a layer-wise hybrid manufacturing strategy, combining GMA-based WAAM with in-situ milling after each deposited layer. This technique ensures both geometric precision and surface quality without significantly extending build time and has demonstrated promising results in tool steel and Inconel systems. A notable collaborative effort among Cranfield University, the University of Manchester, and Northeastern University in China led to the development of a WAAM + inter-pass rolling system, where rolling is performed between deposited layers. This mechanical post-processing effectively introduces strain hardening, refines grain morphology—especially in α+β titanium alloys—and mitigates residual stress accumulation in low-alloy steel. Nevertheless, challenges remain regarding the optimization of inter-pass timing, force control, and the mechanical-thermal coupling effects introduced by these hybrid techniques [83].

While these advances illustrate the growing maturity of WAAM post-processing techniques, the field still lacks standardized evaluation metrics and process windows. Future research must focus on quantitative relationships between post-processing parameters and functional performance (e.g., fatigue life, creep resistance), as well as the integration of adaptive control schemes to dynamically adjust post-processing based on in-situ feedback. Only then can post-processing evolve from a deterministic, trial-and-error stage into an intelligent, model-driven manufacturing step tailored for high-specification industrial components.

## 4. Common Defects and Improvement Methods of WAAM

Although arc additive manufacturing has many advantages, there are still some shortcomings that require our attention and need to be resolved.

### 4.1. Porosity

**Figure 8:**
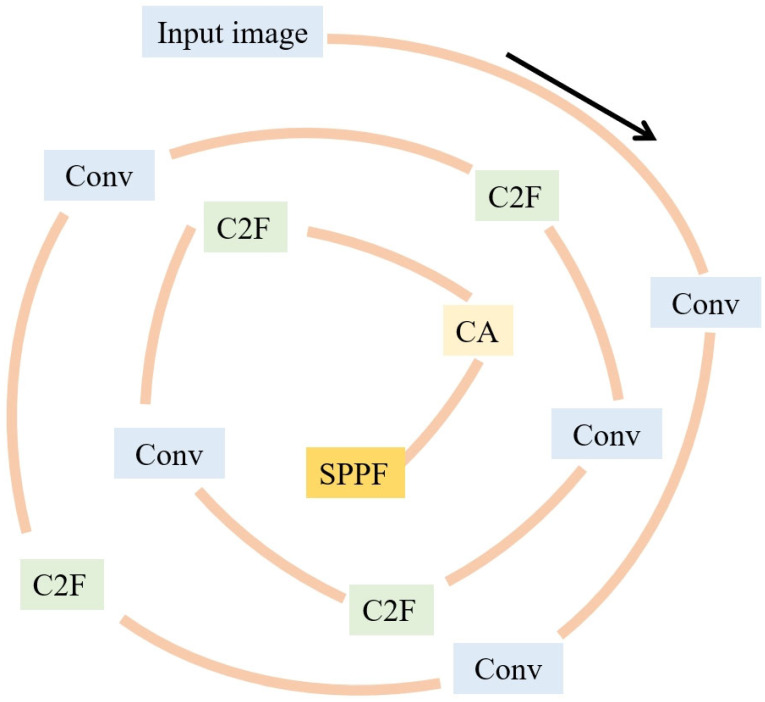
YOLOv8 backbone network.

**Figure 9:**
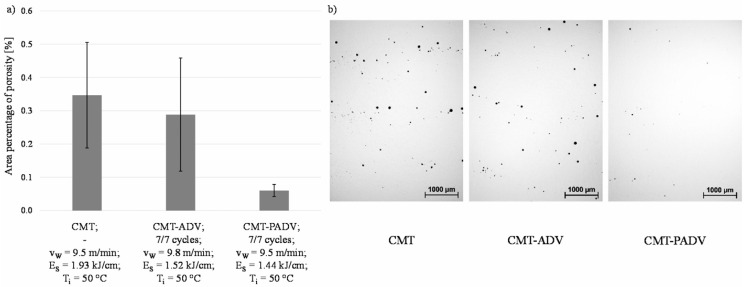
(**a**) Area percentage of porosity in the multilayer walls as a function of the arc mode and (**b**) porosity analysis [89].

Pores are a common defect in AM, which have a serious impact on the mechanical properties of structural parts. An increase in porosity not only significantly reduces the mechanical properties and fatigue strength of structural components but may also lead to defects such as cracking [84,85]. Therefore, it is essential to monitor pore formation during the WAAM process. Pores caused by lack of fusion during deposition are typically irregular in shape rather than spherical. First, increasing the flow rate of pure argon shielding gas can effectively reduce the formation of pores in aluminum alloy additive manufacturing. Subsequently, the pore diameters are statistically classified under four different CMT processes. The results show that most of the pores have a diameter of 10–50 μm. Compared with the other three modes, the traditional CMT mode produces the most pores, and the diameter of some pores exceeds 100 μm. The CMT-P process significantly reduces the number and size of pores, the CMTADV process further reduces the number of pores, and the CMT-PADV process almost eliminates pores. In addition, the additive forming test for 2319 aluminum alloy was carried out by the AC-GTAW process, and the influence of factors such as heat input, working environment (air, argon filling), and wire feeding speed on the internal pores of aluminum alloy forming parts was studied and analyzed. The results indicate that during the AC-GTAW additive manufacturing process of 2319 aluminum alloy, internal porosity defects can be effectively controlled by regulating three key factors: heat input, working environment, and wire feeding speed. Among the three factors, the heat input has the greatest impact on pore defects, and controlling the heat input can effectively reduce the number and size of pores. When heat input is properly controlled, the use of an argon shielding environment can significantly reduce porosity defects in the AC-GTAW WAAM process. Additionally, appropriate regulation of wire feeding speed further contributes to the reduction of porosity in 2319 aluminum alloy components [86,87]. Wei Wang et al. [88] established a defect detection technology based on deep learning and machine vision to inspect the additive manufacturing process. The researchers proposed a YOLOv8 algorithm for training and recognizing defect images. Figure 8 shows the YOLOv8 improved the backbone network. A dataset was then formulated accordingly. After testing, it was found that the improved YOLOv8 achieved an accuracy of 91.7% at a rate of 71.9 frames per second. This level of accuracy enables dynamic monitoring of multi-material processing and more complex structural manufacturing processes. Maximilian Gierth et al. [89] prepared AlMg5Mn multilayer structures by a variety of processes, and the measured porosity is shown in Figure 9. The highest porosity was 0.347% for the standard CMT process, and the porosity was 0.2885 for the CMT-ADV arc process. In contrast, the lowest porosity was 0.06% for the CMT-PADV arc process.

### 4.2. Residual Stress and Strain

Residual stress and strain are inherent defects of the WAAM process and, like other additive manufacturing processes, cannot be avoided. The primary cause of residual stress is the spatial temperature gradient resulting from localized heating and cooling during the operation of the heat source. Another contributing factor is the thermal expansion and contraction of the material during the heating and cooling cycle. Moreover, many process parameters have an impact on residual stress and deformation (environmental temperature, constraints, cladding speed, welding current, etc.). At present, there are already methods that can effectively reduce residual stress and deformation [90,91].

**Figure 10:**
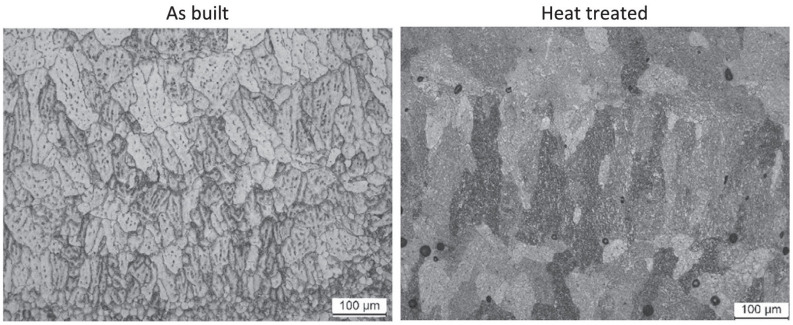
Microstructure before and after heat treatment [92].

(1) Heat treatment: Heat treatment is an effective method for reducing residual stress and enhancing the mechanical properties of components. As shown in Figure 10, Maider Arana et al. [92] found that neither grain size nor morphology of 2319 aluminum alloy annealing samples and heat treatment had any effect on the microstructure, but the coating grains were purer after heat treatment.
(2) Interlayer rolling: Interlayer cold rolling not only reduces residual stress and deformation, but also significantly improves the anisotropy of components. It effectively refines grain structure, mitigates anisotropy and residual stress in additively manufactured components made from titanium alloys, aluminum alloys, and steels. However, the use of the interlayer rolling process is only suitable for simple-shaped components such as straight walls, not for curved surfaces or more complex and irregular components, and the efficiency is low, which has certain limitations [93].

## 5. Application of WAAM Technology

**Figure 11:**
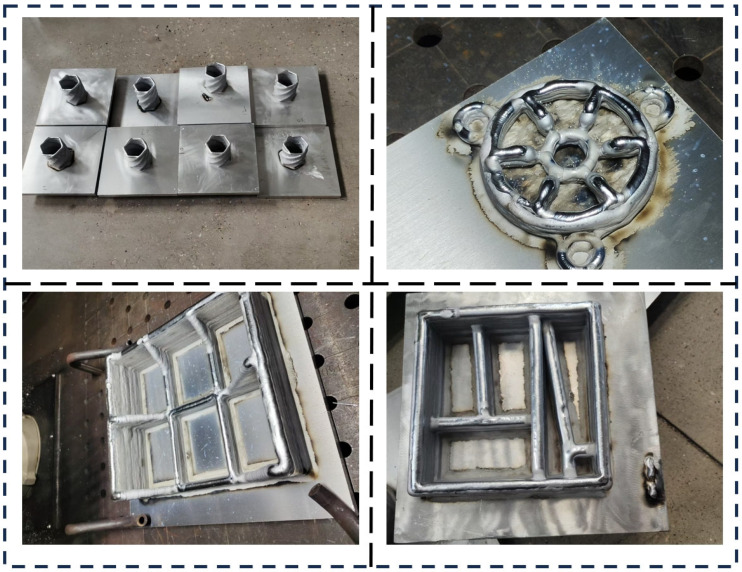
Some actual product applications.

WAAM offers a short overall manufacturing cycle and a high level of flexibility, enabling the benefits of digital, intelligent, and parallel manufacturing. It has demonstrated significant advantages in industries such as automotive, defense, aerospace, and shipbuilding. Additionally, it has found widespread applications in areas like custom chemical production, replacement of traditional manufacturing processes, integration of “material–structure–function,” and mold repair [94,95,96]. Figure 11 shows some actual sample applications. WAAM employs continuous welds as the fundamental structural unit, making it well suited for the rapid prototyping of components such as internal frames, ribs, and wall panels in aircraft applications. At present, the aerospace industry widely utilizes large, monolithic titanium and aluminum alloy components. Cranfield University, located in the UK, stands as a global pioneer in researching the application of titanium alloy WAAM technology. The university has forged significant partnerships with the European Space Agency and Bombardier, enabling the successful production of intricate components, including aircraft wing spars and outer ribs for landing gear support. Currently, the deposition efficiency of titanium alloy using WAAM technology reaches 2 kg/h, with the mechanical properties of the components matching those of forgings. The maximum unidirectional forming size for titanium alloy parts is 1.5 m. Compared with traditional methods, this material significantly reduces the processing cycle and saves up to 69 kg of raw material. Cranfield University in the United Kingdom has also carried out a large number of research studies on the application of aluminum alloy WAAM technology and has trial-produced many aluminum alloy parts. For structural designs based on flexible configuration capabilities, arc additive manufacturing can replace some links in traditional processing and manufacturing. Fushun Donggong Metallurgy in China employs arc additive manufacturing to produce cabin shells, eliminating the need for molds and thereby reducing both product development cycles and overall costs. Nanjing Inigma Co., Ltd. (Nanjing China) uses 921A high-strength weldable alloy structural steel for 3D printing pressure-resistant shells designed for submarines. Traditionally, these components were manufactured through an assembly welding process, which resulted in significant changes to the coarse-grained area of the heat-affected zone under the influence of the welding heat cycle. This heat cycle causes softening in the heat-affected zone, depending on the heat input. However, with arc additive manufacturing, by carefully controlling the heat input and using the corresponding wire material, the printed components achieve a tensile strength of 720 MPa and a yield strength of 620 MPa. In addition, since the raw material of arc additive manufacturing is wire, through multi-filament synergy or composition control, different accumulation parts of components can have different chemical compositions and organizational properties, realizing the integrated manufacturing of dissimilar materials and multifunctional gradient components. Therefore, WAAM is expected to play an important role in the manufacture of multi-material components in the fields of ships, nuclear power, and the chemical industry.

## 6. Conclusions and Outlook

In recent years, WAAM has seen rapid development and has become a key focus in advanced manufacturing, with significant prospects for future growth. In China, the development of wire arc additive manufacturing should be driven by scientific research and aligned with the fundamental needs of national strategic products and key industries. It is essential to focus on the forefront of global advanced manufacturing and industrial development, seizing this historic opportunity for China to achieve a “change lanes and overtake” advancement. Currently, research on arc additive manufacturing encompasses a wide range of areas; this encompasses new process principles, shape control and optimization, material design, structural enhancements, improvements in equipment quality and efficiency, quality inspection and standards, as well as composite additive manufacturing systems. Arc additive manufacturing technology has broad application prospects in the production of large structural parts. Relevant research studies at home and abroad have formed large and more complex metal parts. There are also many universities and enterprises in China that have carried out many test pieces applied in the aerospace field, but they are still in the stage of exploration and research. In addition, the current WAAM technology widely used in the field has problems such as low forming accuracy and uneven microstructure after welding. For the inhomogeneity of the metal structure, the CMT welding power supply can be used to reduce the heat input so as to reduce the heat accumulation generated during the welding process, thereby inhibiting the growth of grains, and finally reducing the formation of microscopic pore defects in the sample. At the same time, combining coaxial melt-pool cameras, infrared thermography, eddy-current probes, and acoustic emission must be integrated with fast decision algorithms so that incipient defects are corrected layer by layer rather than detected post-process.

(1) Development of intelligent arc additive manufacturing equipment with software as the core

At present, arc additive manufacturing equipment is no longer limited to producing products, but also to producing high-quality products. On the one hand, arc additive manufacturing equipment is developing towards the ability to prepare high-precision, high-quality arc additive software, achieving the goal of precise shape control. On the other hand, WAAM equipment is developing towards flexibility and multi-function. Through continuous improvement of equipment and development of intelligent software, the purpose of design and manufacture integration, high precision, process orientation, and global monitoring can be achieved. In addition, arc additive manufacturing equipment is developing towards intelligence. In the process of production and manufacturing, the equipment installation monitoring and measurement system collects relevant information in the manufacturing process, then automatically recognizes the internal cycle and finally achieves rapid manufacturing and intelligent manufacturing.

(2) Forming process optimization and process library establishment

Compared with traditional metal subtractive or stop-material manufacturing technology, arc additive manufacturing has more process parameters, and for different arc additive manufacturing equipment and materials, it is difficult to match the process parameters. Using high-throughput methods, establishing a process database reasonably matched with equipment and materials can greatly reduce the difficulty of matching process parameters. Based on the process library and path planning, the research on the process parameters of the three-dimensional path WAAM technology is explored, the forming process is optimized, and the arc additive manufacturing process is more stable.

(3) Materials and architecture innovation

Beyond conventional steels, aluminum and titanium alloys, future work should target in situ alloying, functionally graded architectures, and metal-matrix composites tailored to WAAM’s thermal signature. Gradient wire feedstocks and arc-assisted directed-energy co-deposition with powders are promising avenues.

(4) Development of integrated system and technology of “additive-subtractive”

At present, the development of the “additive-subtractive” integrated system is in its infancy, mainly focusing on laser sintering, laser cladding + multi-axis milling CNC machine tools. Additionally, arc “additive-subtractive” integrated technology remains relatively limited. Traditional CNC machining “subtractive material technology” has a strong complementary relationship with additive manufacturing. Through the integration of material subtraction technology in the arc additive process, the uneven surface of the formed component can be machined in time, which can solve the problem of low internal and external geometric dimension accuracy and surface finish of arc additive manufacturing metal components. It can not only take advantage of the high efficiency of arc additive forming but also improve its surface precision through milling and cutting, and make up for the disadvantage of poor surface quality of arc additive forming components. Therefore, the development of arc “additive-subtractive” integrated technology is an effective way to solve the dual conflict between metal additive efficiency and precision, and an important technical direction to realize efficient and high-precision overall manufacturing of large complex components.

If these research themes are pursued in a concerted fashion, WAAM can progress from producing near-net-shape demonstrators toward delivering flight-qualified, safety-critical hardware. We believe that integrating intelligent control, rigorous standards, and novel materials will ultimately transform WAAM into a mainstream manufacturing route, fulfilling its promise of agile, large-scale, and cost-effective metal production.

## Figures and Tables

**Table 1:** Comparison of several WAAM processes.

WAAM	Energy Source	Features
GTAW-based	Gas Tungsten Arc Welding	(1) Utilizes a non-consumable tungsten electrode and a separate wire feed system [14]. (2) Excellent control over heat input and weld quality. (3) Typical deposition rate: 1–2 kg/h. (4) Suitable for thin-walled structures and fine-feature parts [15]. (5) Requires complex torch and wire feeding synchronization.
GMAW-based	Gas Metal Arc Welding	(1) Uses a consumable wire electrode directly fed into the molten pool [16]. (2) Higher deposition rates compared to GTAW (typically 3–4 kg/h depending on conditions). (3) Common issues include spatter, porosity, and arc instability. (4) Suitable for medium to large structures requiring faster build rates [17].
CMT-based	Cold Metal Transfer	(1) Advanced GMAW process with reciprocating wire motion for controlled droplet transfer. (2) Very low heat input with virtually no spatter [18]. (3) Typical deposition rate: 2–3 kg/h. (4) High gap-bridging ability and tolerance to positional errors. (5) Well suited for low-distortion, thin-walled components and dissimilar material joining.
PAW-based	Plasma Arc Welding	(1) Utilizes a non-consumable tungsten electrode with a constricted plasma arc and separate wire feed. (2) Higher arc energy density compared to GTAW. (3) Typical deposition rate: 2–4 kg/h. (4) Suitable for high-precision deposition with deeper penetration and finer beads. (5) Requires careful control of plasma gas parameters [19].

**Table 2:** Microhardness and impact results of two kinds of arc additive manufacturing.

	Microhardness (HV)			Impact Test (J)		
	Top	Middle	Bottom	Top	Middle	Bottom
CMT	217.98	222.03	226.18	96	108	112
GMAW	177.45	180.82	184.25	25.8	27.8	26.2

**Table 3:** Comparison of tensile results of two kinds of arc additive manufacturing.

	Tensile Property	UTS (MPa)			YS (MPa)			Elongation (%)		
	Sample	Top	Middle	Bottom	Top	Middle	Bottom	Top	Middle	Bottom
CMT	Observed Value	504	507	512	197	201	205	49.5	52.1	56.5
	Mean Value	507.66			201			52.7		
GMAW	Observed Value	520.6	512.7	504.29	268.6	249.26	251.85	48.98	49.72	49.36
	Mean Value	512.53			256.57			49.35		

## Data Availability

The corresponding author declares on behalf of all authors that all raw data and materials discussed in the study will be freely accessible to any researcher who wants to utilize them for non-commercial research without compromising participant privacy. The corresponding author additionally provides information on where, if applicable, data might be accessed to support the findings described in the paper.
